# Hepatic insulin resistance and muscle insulin resistance are characterized by distinct postprandial plasma metabolite profiles: a cross-sectional study

**DOI:** 10.1186/s12933-024-02188-0

**Published:** 2024-03-16

**Authors:** Anouk Gijbels, Balázs Erdős, Inez Trouwborst, Kelly M. Jardon, Michiel E. Adriaens, Gijs H. Goossens, Ellen E. Blaak, Edith J. M. Feskens, Lydia A. Afman

**Affiliations:** 1 Division of Human Nutrition and Health, Wageningen University, Stippeneng 4, 6708 WE Wageningen, The Netherlands; 2 TI Food and Nutrition (TiFN), Nieuwe Kanaal 9A, 6709 PA Wageningen, The Netherlands; 3 Maastricht Centre for Systems Biology (MaCSBio), Maastricht University, Paul-Henri Spaaklaan 1, 6229 EN Maastricht, The Netherlands; 4 Department of Human Biology, NUTRIM School of Nutrition and Translational Research in Metabolism, Maastricht University Medical Center+, Universiteitssingel 50, 6229 ER Maastricht, The Netherlands

**Keywords:** Insulin resistance, Glucose homeostasis, Postprandial metabolism, Lipid metabolism, Metabolomics, Postprandial lipemia, Lipoproteins, Triglycerides, Meal challenge test, Dyslipidemia

## Abstract

**Background:**

Tissue-specific insulin resistance (IR) predominantly in muscle (muscle IR) or liver (liver IR) has previously been linked to distinct fasting metabolite profiles, but postprandial metabolite profiles have not been investigated in tissue-specific IR yet. Given the importance of postprandial metabolic impairments in the pathophysiology of cardiometabolic diseases, we compared postprandial plasma metabolite profiles in response to a high-fat mixed meal between individuals with predominant muscle IR or liver IR.

**Methods:**

This cross-sectional study included data from 214 women and men with BMI 25–40 kg/m^2^, aged 40–75 years, and with predominant muscle IR or liver IR. Tissue-specific IR was assessed using the muscle insulin sensitivity index (MISI) and hepatic insulin resistance index (HIRI), which were calculated from the glucose and insulin responses during a 7-point oral glucose tolerance test. Plasma samples were collected before (T = 0) and after (T = 30, 60, 120, 240 min) consumption of a high-fat mixed meal and 247 metabolite measures, including lipoproteins, cholesterol, triacylglycerol (TAG), ketone bodies, and amino acids, were quantified using nuclear magnetic resonance spectroscopy. Differences in postprandial plasma metabolite iAUCs between muscle and liver IR were tested using ANCOVA with adjustment for age, sex, center, BMI, and waist-to-hip ratio. *P*-values were adjusted for a false discovery rate (FDR) of 0.05 using the Benjamini–Hochberg method.

**Results:**

Sixty-eight postprandial metabolite iAUCs were significantly different between liver and muscle IR. Liver IR was characterized by greater plasma iAUCs of large VLDL (*p* = 0.004), very large VLDL (*p* = 0.002), and medium-sized LDL particles (*p* = 0.026), and by greater iAUCs of TAG in small VLDL (*p* = 0.025), large VLDL (*p* = 0.003), very large VLDL (*p* = 0.002), all LDL subclasses (all *p* < 0.05), and small HDL particles (*p* = 0.011), compared to muscle IR. In liver IR, the postprandial plasma fatty acid (FA) profile consisted of a higher percentage of saturated FA (*p* = 0.013), and a lower percentage of polyunsaturated FA (*p* = 0.008), compared to muscle IR.

**Conclusion:**

People with muscle IR or liver IR have distinct postprandial plasma metabolite profiles, with more unfavorable postprandial metabolite responses in those with liver IR compared to muscle IR.

**Supplementary Information:**

The online version contains supplementary material available at 10.1186/s12933-024-02188-0.

## Introduction

Overweight, obesity and related metabolic complications and chronic diseases, such as cardiovascular disease (CVD) and type 2 diabetes (T2DM), pose a massive burden to public health [1]. Insulin resistance (IR) is one of the earliest metabolic disturbances that underlies the development of many obesity-related metabolic complications [2]. Apart from its central role in glucose homeostasis, insulin is a major regulator of lipid and protein metabolism. As such, IR is commonly accompanied by lipid and lipoprotein abnormalities, although the causal and temporal relationships of these links are unclear [3].

The pathophysiology of whole-body IR is characterized by great heterogeneity, with inter-individual differences in IR severity in the various metabolic organs, including the liver and skeletal muscle. Tissue-specific IR in liver and skeletal muscle has previously been linked to distinct plasma metabolite and lipidome profiles [4, 5]. These findings may indicate that either the mechanisms causing tissue-specific IR differ between the affected tissues or that tissue-specific IR results in different metabolic disturbances. More specifically, muscle IR has been associated with lower fasting plasma concentrations of lysophosphatidylcholines, while liver IR has been associated with higher fasting plasma levels of triacylglycerols (TAG) and ketogenic amino acids, lower levels of ketone bodies, and higher diacylglycerols (DAG), the latter in women, but not in men [4, 5].

Furthermore, liver IR has been characterized by elevated postprandial total TAG in response to an oral fat load, compared to muscle IR and insulin-sensitive individuals [6]. Changes in postprandial metabolite levels reflect the complex interplay of the production, secretion, and clearance by the various metabolic organs, in particular the liver, adipose tissue, and skeletal muscle. Therefore, postprandial metabolite concentrations may provide more insights into the metabolism and functioning of these key metabolic organs than fasting metabolite levels. Importantly, early metabolic perturbations are more likely to become apparent in the postprandial state, when complex processes in these tissues act to maintain or regain homeostasis. Accordingly, postprandial metabolites, including TAGs, in the circulation are important predictors of risk for future CVD and related metabolic diseases, independent of fasting measures [7–9].

To gain a better understanding of fasting and postprandial metabolism in tissue-specific IR, we compared fasting and postprandial plasma metabolite profiles, including lipoproteins, apolipoproteins, cholesterol, triglycerides, ketone bodies, and amino acids, in response to a high-fat mixed meal in individuals with predominant muscle IR or liver IR.

## Methods

### Study design and participants

This study is a cross-sectional analysis using baseline data from the PERSonalized Glucose Optimization Through Nutritional Intervention (PERSON) study, a two-center, randomized, dietary intervention trial that was conducted from May 2018 until November 2021 at Maastricht University Medical Center + (MUMC+) and Wageningen University (WUR) in the Netherlands. The design and methodology have been described in detail previously [10]. The trial was performed in line with the principles of the Declaration of Helsinki, approved by the Medical Ethical Committee of the MUMC + (NL63768.068.17), and registered at ClinicalTrials.gov (NCT03708419). All participants gave written informed consent.

Participants were recruited via a volunteer database, flyers, and local newspaper and online media advertisements. Inclusion criteria were: age 40–75 years, BMI 25–40 kg/m^2^, body weight stability for at least three months (no weight gain or loss > 3 kg), and tissue-specific IR, characterized as predominant muscle or liver IR. Exclusion criteria included pre-diagnosis of T2DM, diseases or medication use that affect glucose or lipid metabolism (e.g. pheochromocytoma, Cushing's syndrome, acromegaly, or chronic use of fibrates, thiazolidinediones, or NSAIDs), major gastrointestinal disorders, history of major abdominal surgery, uncontrolled hypertension, smoking, alcohol consumption > 14 units/wk, and > 4 h/wk moderate-to-vigorous physical activity (metabolic equivalents [METs] > 3.0). Using statins was not an exclusion criterion in the original trial because we did not expect interference with the primary study outcomes. However, statin users were excluded from the current analysis due to statins’ effects on fasting and postprandial cholesterol and triglycerides [11, 12]. Data on demographics, medical history, family history of diabetes (≥ 1 first-degree relative with diabetes), and medication use and lifestyle were collected by questionnaire.

### Tissue-specific insulin resistance

Details on the assessment of eligibility have been described previously [13]. Tissue-specific IR was assessed at screening and baseline using the plasma glucose and insulin concentrations during a 7-point oral glucose tolerance test (OGTT). After an overnight fast, participants ingested 200 mL of a 75 g glucose drink (Novolab) within 5 min. Blood samples were collected from the antecubital vein via intravenous cannula before (T = 0 min) and after ingestion of the glucose drink (T = 15, 30, 45, 60, 90, and 120 min). Plasma glucose and insulin concentrations were quantified by enzymatic assay or enzyme-linked immunoassay (ELISA), respectively, and used for calculation of the muscle insulin sensitivity index (MISI) and hepatic insulin resistance index (HIRI) [14, 15]. MISI was calculated as: *(dGlucose/dt ÷ [mean insulin in pmol/L])*, where *dGlucose/dt* is the rate of decay of plasma glucose concentration (mmol/L) during the OGTT, calculated as the slope of the least square fit to the decline in plasma glucose concentration from peak to nadir [14, 15]. HIRI was calculated as: *([glucose*_*AUC 0–30*_ in mmol/L*h] × *[insulin*_*AUC 0–30*_ in pmol/L*h]). Tertile cut-offs for MISI and HIRI from a previous study with a similar study population [16] were used to identify individuals with predominant muscle IR or liver IR: individuals in the lowest tertile of MISI and lowest or middle tertile of HIRI were classified as predominant muscle IR, while individuals in the highest tertile of HIRI and middle or highest tertile of MISI were classified as predominant liver IR. Baseline measurements were performed within three months after screening. In this analysis, MISI and HIRI from the screening and baseline measurements were averaged.

The Matsuda index, a measure of whole-body insulin sensitivity, was calculated using glucose and insulin values from time points 0, 30, 60, 90, and 120 min: *(10,000 ÷ square root of [fasting plasma glucose in mmol/L* × *fasting insulin in mU/L]* × *[mean glucose in mmol/L x mean insulin in mU/L])* [17]. Glucose status was defined according to WHO criteria [18]: normal glucose tolerance (NGT), fasting glucose < 6.1 mmol/L and 2-h glucose < 7.8 mmol/L; impaired fasting glucose (IFG), fasting glucose 6.1 – 6.9 mmol/L and 2-h glucose < 7.8 mmol/L; impaired glucose tolerance (IGT), fasting glucose < 6.1 mmol/L and 2-h glucose 7.8–11.0 mmol/L; combined IFG/IGT, fasting glucose 6.1–6.9 mmol/L and 2-h glucose 7.8–11.0 mmol/L; T2DM, fasting glucose ≥ 7.0 mmol/L and/or 2-h glucose ≥ 11.1 mmol/L.

### High-fat mixed-meal test

After a 12-h overnight fast, participants visited the facilities for a high-fat mixed-meal test. The evening before the visit, participants consumed a standardized low-fat pasta meal (30% of energy intake [en%] fat, 49 en% carbohydrates [CHO], 21 en% protein; 1560–2460 kJ, depending on estimated energy requirements), and they were instructed to refrain from alcohol and vigorous physical activities for three days before the visit. The liquid high-fat mixed meal was prepared in the metabolic kitchen using ice cream, full-fat milk, whipped cream, and sugar. It contained 49 g fat (33 g saturated fat [SFA]), 48 g CHO, and 12 g protein (Table 1).

**Table 1:** Ingredients and nutrient composition of the high-fat mixed meal

	Ice cream	Full-fat milk	Whipped cream	Sugar	Total per meal
Amount per meal, g	150	125	70	5	350
Energy, kJ	1388	348	973	85	2793
Protein, g	5.6	4.5	1.5	0	11.6
Fat, g	19.5	4.5	24.6	0	48.6
Saturated fat, g	12.8	3.1	17.5	0	33.4
Carbohydrates, g	34.5	5.9	2.2	5.0	47.5
Sugar, g	31.5	5.9	2.2	5.0	44.5

An intravenous cannula was inserted in the antecubital vein, and a fasting blood sample was drawn at least 30 min after insertion. Participants consumed the meal within 5 min. Postprandial blood samples were drawn at T = 30, 60, 90, 120, 180, and 240 min.

Glucose and insulin levels were measured in EDTA plasma from timepoints 0, 30, 60, 120, 180, and 240 min by enzymatic assay or ELISA, respectively. Fasting plasma non-esterified fatty acids (NEFA) and fasting serum TAG, total cholesterol, and high-density lipoprotein (HDL) cholesterol were quantified with enzymatic assays. Hypertriglyceridemia was defined as fasting serum triglyceride ≥ 1.7 mmol/L. The inflammatory marker C-reactive protein (CRP) was measured in fasting plasma with a Luminex immunoassay.

The homeostasis model assessment of insulin resistance (HOMA-IR) was calculated as *(fasting glucose in mmol/L* × *fasting insulin in mU/L) ÷ 22.5,* and HOMA of β-cell function (HOMA-β) was calculated as *(20* × *fasting insulin in mU/L) ÷ (fasting glucose in mmol/L – 3.5)* [19]. Adipose tissue IR (Adipo-IR) was estimated by calculating (*fasting insulin in pmol/L x fasting NEFA in mmol/L).*

### Fasting and postprandial metabolite profile

Metabolite concentrations were quantified in plasma samples from T = 0, 30, 60, 120, and 240 min by the Nightingale high-throughput nuclear magnetic resonance (NMR) metabolomics platform (Nightingale Health Ltd., Helsinki, Finland) [20]. This platform provides quantitative data on 164 metabolites, including 14 lipoprotein subclasses, their lipid concentrations and composition, apolipoprotein A-I (ApoA) and B (ApoB), major fatty acids, (branched-chain) amino acids (BCAA), glycolysis-related measures, and ketone bodies. In addition, it provides data on three lipoprotein sizes (very-low-density lipoprotein [VLDL], low-density lipoprotein [LDL], and HDL diameter) and 82 relative measures (i.e. percentages, ratios). We used clinically measured plasma glucose rather than NMR-measured glucose and excluded the measure ‘Unsaturation’, assessing a total of 247 metabolic measures.

The postprandial net incremental area under the curves (iAUC) were calculated using the trapezoid method. For the calculation of iAUCs, metabolite curves from participants were excluded if values of ≥ 2 time points were missing (n = 2) and/or if the last (T = 240 min) value was missing (n = 5). For metabolite curves with one missing value at 30–120 min, the missing values were imputed with the weighted metabolite average of the two closest time points of that particular metabolite of that participant (n = 13).

### Anthropometrics, body composition and ectopic fat

Waist and hip circumference were measured in duplicate using a non-flexible measuring tape. Whole-body and regional fat mass (i.e. android and gynoid fat mass) were assessed using dual-energy X-ray absorptiometry (DXA) (WUR, Lunar Prodigy, GE Healthcare; MUMC+, Discovery A, Hologic). Intrahepatic lipid content was quantified after a ≥ 2-h fast with a 3T magnetic resonance imaging (MRI) scanner using proton magnetic resonance spectroscopy (^1^H-MRS) (WUR) or a 6-min whole-body MRI scan protocol and automated image analysis (MUMC +) (AMRA Medical AB, Linköping, Sweden). Visceral adipose tissue (VAT) volume was also quantified in MUMC + participants from the MRI. Details of these methods have been described previously [10].

### Habitual dietary intake and physical activity

Habitual dietary intake was assessed with a validated 163-item semi-quantitative food frequency questionnaire (FFQ) [21]. Diet quality was assessed with the Dutch Healthy Diet index 2015 (DHD15-index) [22], which is a score between 0 (no adherence) and 150 (complete adherence) that reflects adherence to the Dutch dietary guidelines. Self-reported habitual physical activity was assessed with the Baecke questionnaire [23].

### Statistical analyses

Baseline characteristics were compared between IR phenotypes and between men and women using an independent t-test for normally distributed numerical data, a Mann–Whitney test for non-normally distributed numerical data, and using Fisher’s exact test for categorical data.

Differences in fasting plasma metabolite levels and postprandial metabolite iAUCs between muscle IR and liver IR were tested using ANCOVA with adjustment for age, sex, study center, BMI, and waist-to-hip ratio. Associations between MISI/HIRI and fasting plasma metabolites and postprandial metabolite iAUCs were tested using linear regression analyses with adjustment for age, sex, study center, BMI, waist-to-hip ratio, and HIRI/MISI. Since sex-specific associations between tissue-specific IR and fasting plasma metabolites have been reported previously [4, 5], we tested for effect modification by sex by testing interactions between IR phenotype or MISI/HIRI and sex. For the linear regression analyses and ANCOVA, fasting metabolites, metabolite iAUCs, MISI and HIRI were log-transformed (log2) and autoscaled to allow for direct comparison of effect sizes. *P*-values were adjusted for a false discovery rate (FDR) of 0.05 using the Benjamini Hochberg method [26].

In addition, because the iAUC may not fully capture postprandial metabolite dynamics as iAUCs may be similar for postprandial curves with a different shape, we performed multivariate analysis to compare the shapes of the metabolite responses between IR phenotypes via RM-ASCA +  [24, 25]. Details can be found in Additional file 1.

## Results

Data on plasma metabolomics were available from 230 participants: 142 individuals with muscle IR and 88 with liver IR. Sixteen participants were excluded from analyses due to statin use (muscle IR, 7.7%, n = 11; liver IR, 5.7%, n = 5), resulting in 131 individuals with muscle IR and 83 individuals with liver IR that were included in the analyses.

### Baseline characteristics

Table 2 shows anthropometrics, body composition, glucose homeostasis, cardiometabolic parameters, medical history, and lifestyle factors according to IR phenotype. Sex-stratified characteristics according to IR phenotype are reported in Additional file 2: Table S1. The participants’ mean (± SD) age was 60 ± 8 years, and 61% were women. Individuals with liver IR had higher BMI and waist circumference, lower plasma CRP and borderline lower liver fat (WUR subgroup) than those with muscle IR. In women only, VAT mass was higher in liver compared to muscle IR. In line with the calculations of MISI and HIRI, fasting plasma glucose and insulin levels were higher in liver IR, which was due to differences in women only, and plasma glucose and insulin two hours after oral glucose load were higher in muscle IR, which was due to differences in men only. Body fat percentage and whole-body insulin sensitivity—as determined by Matsuda index—were not different between muscle and liver IR in the total study population, but Matsuda index was lower in liver compared to muscle IR in women.

**Table 2:** Demographic, clinical, and metabolic characteristics of the study population according to IR phenotype

	Muscle IR (n = 131)	Liver IR (n = 83)	*p*
Age, years	60 ± 8	59 ± 7	0.33
Women, n (%)	84 (64.1%)	46 (55.4%)	0.25
BMI, kg/m^2^	29.6 ± 3.3	30.7 ± 3.8	**0.033**
Waist circumference, cm	101.0 ± 8.8	103.8 ± 10.8	**0.034**
**Body composition**			
Body fat, %	38.3 ± 7.5	37.0 ± 7.0	0.20
Android fat, %	10.2 ± 1.8	10.2 ± 1.7	0.90
Gynoid fat, %	15.9 ± 2.1	15.7 ± 2.0	0.44
VAT, L^a^	5.4 ± 2.2	6.1 ± 2.1	0.12
VAT, cm^2 b^	163 [119, 226]	176 [135, 200]	0.49
Liver fat, %^a^	4.9 [2.6, 9.9]	5.7 [3.3, 14.8]	0.29
Liver fat, %^b^	4.2 [1.4, 8.2]	2.4 [1.0, 5.2]	0.06
**Glucose homeostasis**			
Fasting glucose, mmol/L	5.3 [5.0, 5.6]	5.5 [5.1, 5.8]	**0.014**
OGTT 2-h glucose, mmol/L	6.5 [5.4, 7.7]	5.7 [4.8, 6.9]	**0.002**
Fasting insulin, pmol/L	44.7 [37.2, 60.1]	50.5 [41.0, 69.3]	**0.012**
OGTT 2-h insulin, pmol/L	403.3 [264.5, 585.6]	309.5 [192.8, 571.6]	**0.017**
HOMA-IR, AU	1.7 [1.3, 2.1]	1.9 [1.3, 2.5]	0.08
HOMA-β, AU	76.4 [62.5, 96.1]	82.4 [64.5, 98.8]	0.54
Matsuda index, AU	4.8 [3.5, 6.7]	4.2 [3.0, 6.5]	0.18
Adipo-IR, AU	21.7 [15.0, 30.0]	23.7 [16.1, 39.4]	0.14
MISI, AU	0.096 [0.068, 0.130]	0.135 [0.104, 0.183]	** < 0.001**
HIRI, AU	356 [284, 432]	601 [467, 716]	** < 0.001**
Glucose status, n (%)			0.25
NGT	101 (77.1%)	65 (78.3%)	
IGT	18 (13.7%)	6 (7.2%)	
IFG	2 (1.5%)	5 (6.0%)	
IGT + IFG	8 (6.1%)	5 (6.0%)	
T2DM	2 (1.5%)	2 (2.4%)	
**Cardiometabolic parameters**			
Fasting serum TAG, mmol/L	1.3 [1.0, 1.7]	1.5 [1.0, 1.9]	0.22
Fasting hypertriglyceridemia, n (%)	37 (28.2%)	30 (36.1%)	0.29
Serum HDL cholesterol, mmol/L	1.3 ± 0.3	1.3 ± 0.3	0.34
Serum total cholesterol, mmol/L	5.4 ± 1.0	5.5 ± 1.0	0.39
Fasting plasma NEFA, mmol/L	0.50 ± 0.17	0.48 ± 0.16	0.42
Plasma CRP, mg/L	1.4 [0.6, 2.5]	1.0 [0.5, 2.1]	**0.045**
**Medical history**			
Medication use, n (%)			
Antidepressants	7 (5.3%)	6 (7.2%)	0.57
Antihypertensives	24 (18.3%)	10 (12.0%)	0.25
Anti-inflammatory	15 (11.5%)	4 (4.8%)	0.14
Other	41 (31.3%)	24 (28.9%)	0.76
Family history of DM, n (%)	29 (22.1%)	18 (21.7%)	1.00
**Lifestyle factors**			
DHD2015-index, score	85.7 ± 15.4	81.3 ± 15.1	**0.044**
Habitual fat intake, en%	37.3 ± 5.5	37.2 ± 6.5	0.87
Habitual SFA intake, en%	13.6 ± 2.5	13.9 ± 3.1	0.48
Habitual sugar intake, en%	19.8 ± 5.1	19.2 ± 6.7	0.44
Habitual alcohol consumption, g	5.0 [1.7, 10.3]	6.1 [1.1, 14.7]	0.47
Habitual physical activity, Baecke score	8.2 ± 1.1	8.4 ± 1.3	0.35

Differences between IR phenotypes were assessed using independent t-test for normally distributed numerical data (mean ± SD), Mann–Whitney test for non-normally distributed numerical data (median [25th percentile, 75th percentile], and using Fisher’s exact test for categorical data (n [%]). *P*-values < 0.05 are highlighted in bold

*BMI* body mass index, *VAT* visceral adipose tissue, *OGTT* oral glucose tolerance test, *HOMA-IR* homeostatic model assessment of insulin resistance, *HOMA-β* homeostatic model assessment of β-cell function, *Adipo-IR* adipose tissue insulin resistance, *MISI* muscle insulin sensitivity index, *HIRI* hepatic insulin resistance index, *NGT* normal glucose tolerant, *IGT* impaired glucose tolerance, *IFG* impaired fasting glucose, *T2DM* type 2 diabetes mellitus, *TAG* triacylglycerol, *NEFA* non-esterified fatty acids, *CRP* C-reactive protein, *DHD2015-index* Dutch Healthy Diet 2015 index, *SFA* saturated fatty acids

### Glucose and insulin responses to the mixed meal in liver IR and muscle IR

**Fig. 1:**
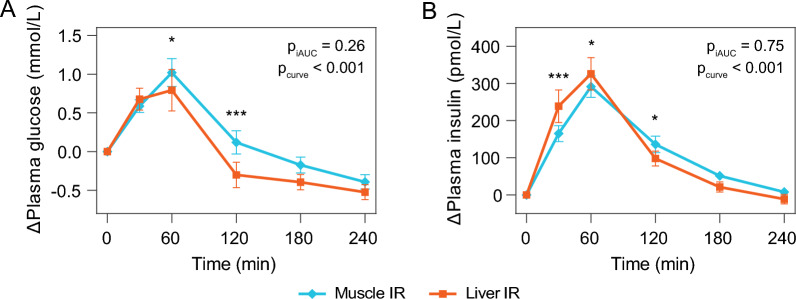
Plasma glucose (**A**) and insulin (**B**) responses to consumption of the high-fat mixed meal in individuals with liver IR or muscle IR. Responses were defined as change from fasting (value at postprandial timepoint—fasting value), and data are shown as means with 95% confidence intervals. Differences between liver IR and muscle IR were tested using linear mixed-effects models with adjustment for age, sex, center, and BMI. Significant LSD post-hoc pairwise comparisons per timepoint are denoted with *(*p* < 0.05) or ***(*p* < 0.001)

After consumption of the mixed meal, plasma glucose levels in muscle IR were higher one and two hours post-meal compared to liver IR (*p*_curve_ < 0.001) (Fig. 1A). Plasma insulin levels were higher in liver compared to muscle IR in the first hour, and were lower two hours post-meal (*p*_curve_ < 0.001). Total iAUCs of glucose (*p*_iAUC_ = 0.26) and insulin (*p*_iAUC_ = 0.75) did not differ between the IR phenotypes (Fig. 1B).

### Fasting plasma metabolites in liver IR and muscle IR

We compared fasting plasma metabolites between individuals with muscle IR and liver IR and examined associations of MISI and HIRI with fasting plasma metabolite concentrations (Additional file 2: Table S2). All analyses with a significant interaction between IR phenotype or MISI/HIRI and sex were performed with stratification for sex.

None of the 164 absolute plasma metabolite concentrations were significantly different between individuals with muscle or liver IR after FDR correction. One of the 82 relative fasting metabolite measures showed a trend for a difference between muscle and liver IR in the fasting state (Additional file 1: Figs. S1–2), and a significant sex interaction was observed for this metabolite. The VLDL, LDL, and HDL particle sizes did not differ between IR phenotypes in the fasting state. The relative fasting metabolite measure that tended to differ between the IR phenotypes was the percentage of saturated fatty acid of total plasma FA (SFA%), which was higher in muscle IR (geometric mean 33.5%, 95% CI 33.2 to 33.9) compared to liver IR (32.5%, 32.1 to 32.9; *p* = 0.072), in men only (Additional file 1: Fig. S2 and Additional file 2: Table S2). Additional adjustment for habitual dietary intake of fat, SFA, linoleic acid (LA), or CHO did not affect this finding (data not shown).

### Associations between MISI and HIRI with fasting plasma metabolites

Both MISI and HIRI were not significantly associated to any of the 164 absolute fasting plasma metabolite concentrations after FDR correction. Out of the 82 relative metabolite measures, MISI was associated with one metabolite measure and HIRI with none. Both indices were not associated with the three particle sizes. The one metabolite that MISI was positively associated with was the percentage of LA (LA%) of total plasma FA, but only in women (std. β 0.320, 0.167 to 0.490, *p* = 0.024) (Additional file 1: Fig. S2, Additional file 2: Table S2). Additional adjustment for habitual dietary intake of fat, SFA, LA, or carbohydrates did not affect this association (data not shown).

### Postprandial metabolite responses in liver IR and muscle IR

Next, we compared the postprandial plasma metabolite responses between individuals with muscle IR and liver IR by testing differences in the iAUCs and postprandial curves after the mixed meal between the IR phenotypes. In addition, we examined associations of MISI and HIRI with postprandial plasma metabolite iAUCs. Analyses with a significant interaction between IR phenotype or MISI/HIRI and sex were performed with stratification for sex. All results can be found in Additional file 2: Tables S3-S4.

Forty-four out of 164 absolute metabolite iAUCs differed significantly between muscle and liver IR, of which nine only in women (Additional file 1: Fig. S1). Twenty-four of the 82 relative metabolite iAUCs differed significantly between muscle and liver IR, of which one only in women (Additional file 1: Fig. S1). VLDL, LDL, and HDL particle sizes did not differ postprandially between IR phenotypes.

**Fig. 2:**
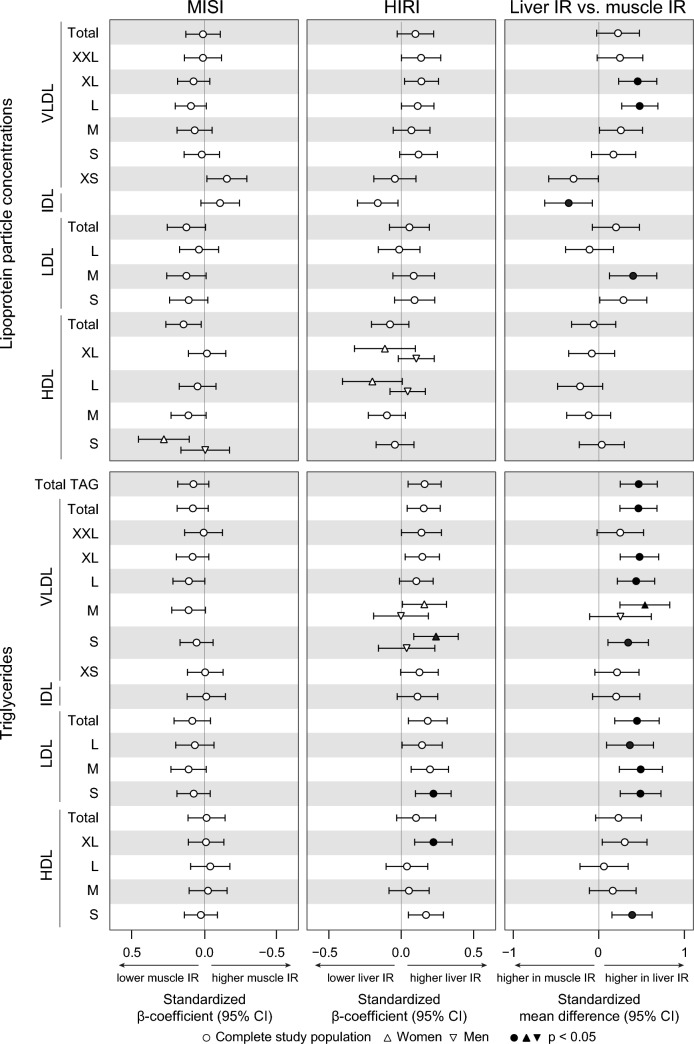
Postprandial (iAUC) lipoprotein particle concentrations and triglycerides (TAG) in muscle and liver IR. Left: associations of MISI with plasma metabolite iAUCs. Middle: associations of HIRI with plasma metabolite iAUCs. Right: plasma metabolite iAUCs in muscle compared to liver IR. Associations between MISI/HIRI and plasma metabolites were tested using linear regression analyses with adjustment for age, sex, center, BMI, waist-to-hip ratio, and HIRI/MISI. Differences between muscle and liver IR were tested using ANCOVA with adjustment for age, sex, center, BMI, and waist-to-hip ratio. *P*-values were adjusted for a false discovery rate (FDR) of 0.05 using the Benjamini Hochberg method

Out of the 44 absolute metabolite iAUCs that differed between the IR phenotypes, a majority was larger in liver compared to muscle IR. Postprandial iAUCs of XL VLDL (*p*_iAUC_ = 0.002), L VLDL (*p*_iAUC_ = 0.004), and M LDL particle concentrations (*p*_iAUC_ = 0.026), as well as their TAG and cholesterol content (all *p* < 0.03) were higher in liver compared to muscle IR, while postprandial intermediate-density lipoprotein (IDL) particle concentrations (*p*_iAUC_ = 0.047) were lower in liver compared to muscle IR (Fig. 2; Additional file 1: Figs. S3–S4). Furthermore, iAUCs of postprandial total TAG (*p*_iAUC_ = 0.004), and TAG in total VLDL (*p*_iAUC_ = 0.003), S VLDL (*p*_iAUC_ = 0.025), total LDL (*p*_iAUC_ = 0.009), all LDL subclasses (all *p*_iAUC_ < 0.05), and S HDL (*p*_iAUC_ = 0.011) were higher in liver compared to muscle IR (Figs. 2; Additional file 1: Fig. S4).

**Fig. 3:**
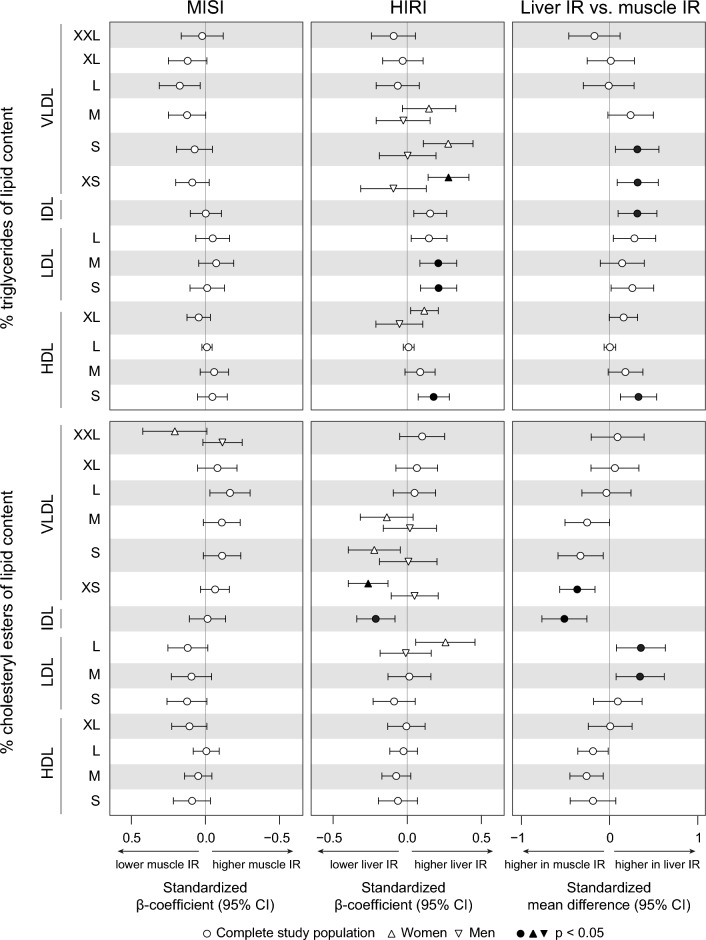
Postprandial (iAUC) TAG and CE content expressed as percentages of total lipoprotein lipid content in muscle and liver IR. Left: associations of MISI with plasma metabolite iAUCs. Middle: associations of HIRI with plasma metabolite iAUCs. Right: plasma metabolite iAUCs in muscle compared to liver IR. Associations between MISI/HIRI and plasma metabolites were tested using linear regression analyses with adjustment for age, sex, center, BMI, waist-to-hip ratio, and HIRI/MISI. Differences between muscle and liver IR were tested using ANCOVA with adjustment for age, sex, center, BMI, and waist-to-hip ratio. *P*-values were adjusted for a false discovery rate (FDR) of 0.05 using the Benjamini Hochberg method

As for the lipid composition of lipoproteins, the iAUC of the postprandial percentage of TAG (TAG%) was higher and that of cholesterol esters (CE%) was lower in XS VLDL and IDL in liver compared to muscle IR (all *p*_iAUC_ < 0.05). In addition, the iAUCs of postprandial TAG% in S HDL and CE% in L and M LDL were higher in liver compared to muscle IR (Figs. 3; Additional file 1: Fig. S4).

**Fig. 4:**
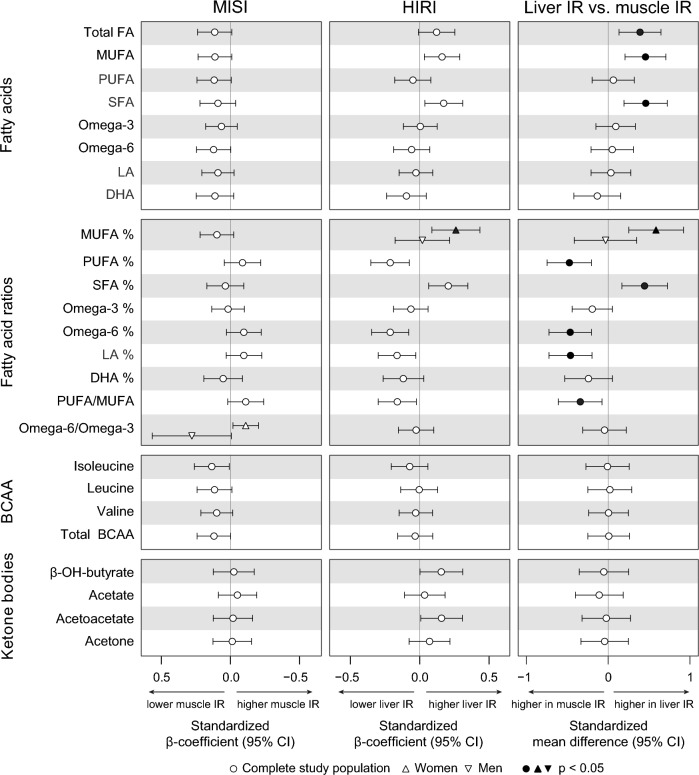
Postprandial (iAUC) fatty acids, fatty acid ratios, ketone bodies and branched-chain amino acids (BCAA) in muscle and liver IR. Left: associations of MISI with plasma metabolite iAUCs. Middle: associations of HIRI with plasma metabolite iAUCs. Right: plasma metabolite iAUCs in muscle compared to liver IR. Associations between MISI/HIRI and plasma metabolites were tested using linear regression analyses with adjustment for age, sex, center, BMI, waist-to-hip ratio, and HIRI/MISI. Differences between muscle and liver IR were tested using ANCOVA with adjustment for age, sex, center, BMI, and waist-to-hip ratio. *P*-values were adjusted for a false discovery rate (FDR) of 0.05 using the Benjamini Hochberg method

Postprandial plasma fatty acid (FA) profiles also differed between the IR phenotypes, with higher postprandial iAUCs of total FA (*p*_iAUC_ = 0.018), total monounsaturated fatty acids (MUFA) (*p*_iAUC_ = 0.005) and total SFA (*p*_iAUC_ = 0.007) in liver compared to muscle IR (Fig. 4 and Additional file 1: Fig. S5). Postprandial total SFA% iAUC was higher (*p*_iAUC_ = 0.013), and total polyunsaturated fatty acid (PUFA)% iAUC was lower in liver compared to muscle IR (*p*_iAUC_ = 0.008), as were iAUCs of the percentages of the PUFAs omega-6 FA (*p*_iAUC_ = 0.007), and LA (*p*_iAUC_ = 0.008) (Fig. 4 and Additional file 1: Fig. S5). Additional adjustment for habitual dietary intake of fat, SFA, or LA did not affect these results (data not shown).

Ten of the 68 observed differences in postprandial metabolite iAUCs between the IR phenotypes were found in women only. These include higher iAUCs of postprandial TAG in M VLDL (*p*_iAUC_ = 0.007), cholesterol and cholesteryl esters in M LDL (*p*_iAUC_ = 0.010 and *p*_iAUC_ = 0.006, respectively) and S LDL (*p*_iAUC_ = 0.021 and *p*_iAUC_ = 0.010, respectively) in liver compared to muscle IR (Additional file 1: Fig. S3). In addition, postprandial MUFA% iAUC was higher in liver compared to muscle IR (*p*_iAUC_ = 0.008).

Postprandial iAUCs of (branched-chain) amino acids, ketone bodies, glycolysis-related metabolites, or other metabolites did not differ between muscle IR and liver IR (Fig. 4).

**Fig. 5:**
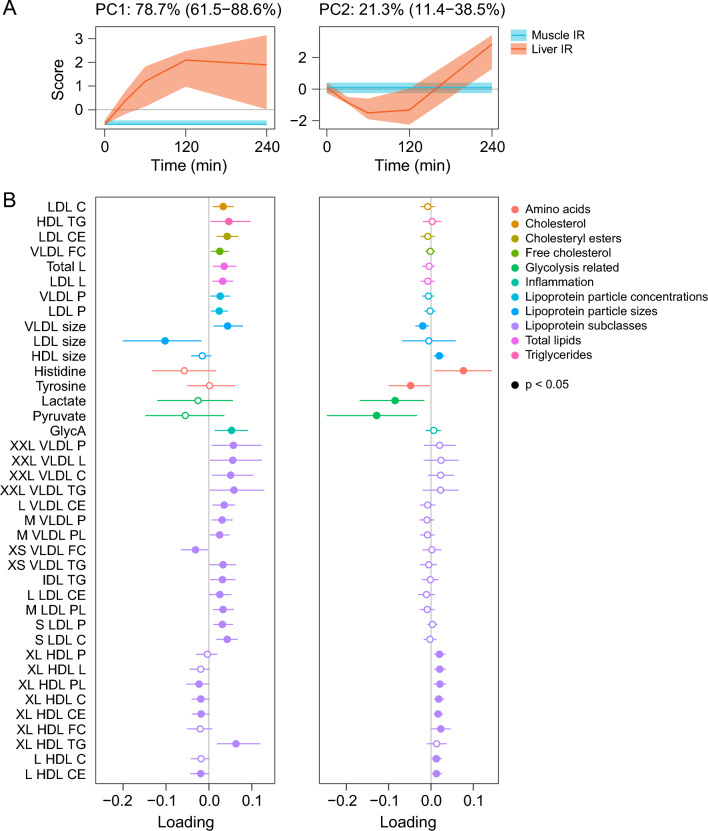
Score trajectory (**A**) and loading (**B**) plots of the difference in postprandial metabolite responses in liver IR compared to muscle IR using RM-ASCA + analysis. **A** Scores containing the predominant patterns over time for PC1 and PC2. Bootstrapped 95% confidence intervals are shown as shaded area for the scores. **B** Loading plots of a set of metabolites that includes those with a significant loading for PC1 and/or PC2, excluding those with a significantly different iAUC between IR phenotypes and excluding relative lipoprotein lipid composition (percentages). The loadings show the association of the scores with the metabolite responses. A positive loading indicates a positive association between the metabolite response and the score (pattern) as shown in this figure, while a negative loading indicates a negative association between the metabolite response and the score (pattern) as shown in this figure, i.e. the inverse of the depicted pattern. Bootstrapped 95% confidence intervals are shown as error bars for the loadings. Filled dots indicate *p* < 0.05

We additionally performed multivariate analysis via RM-ASCA + to investigate differences in overall postprandial metabolite curve shapes between IR phenotypes. PCA analysis of the RM-ASCA + results was used to summarize the differences in postprandial metabolite response in liver IR as compared to muscle IR. Two patterns were identified: PC1 and PC2 (Fig. 5A). PC1 (explained variance [95% CI] 78.7% [61.5–88.6%]) reflects a continuously larger postprandial increase in metabolite concentrations in liver compared to muscle IR in case of positive loadings, and the inverse pattern in case of negative loadings (i.e. a continuously larger postprandial decrease in metabolite concentrations in liver compared to muscle IR). This pattern was observed for 109 metabolites, of which 44 did not differ between IR phenotypes when comparing iAUCs between IR phenotypes, and were thus only identified as different when comparing curve shapes. Metabolites with positive loadings included XXL VLDL particles and chylomicrons and their lipid content, TAG in IDL and HDL particles, VLDL size, and glycoprotein acetylation (GlycA), indicating larger postprandial increases in liver compared to muscle IR. Metabolites with negative loadings include LDL size, indicating the inverse pattern (Fig. 5B and Additional file 1: Fig. S6). PC2 (21.3% [11.4–38.5%] explained variance) reflects a larger decrease at 1–2 h postprandially, followed by a steeper increase at 4 h postprandially in liver compared to muscle IR in case of positive loadings, and the inverse pattern in case of negative loadings. This pattern was observed for 41 metabolites, of which 22 were only identified to be different when comparing postprandial curve shape as they did not have different iAUCs between IR phenotypes. Metabolites with positive loadings included histidine, XL HDL particle concentrations and their lipid content, HDL size, and the cholesterol and cholesteryl ester fraction of VLDL particles (Fig. 5B and Additional file 1: Fig. S6). Metabolites with negative loadings included pyruvate, lactate, tyrosine, and the TAG fraction of VLDL particles. A plot with all metabolites that significantly contributed to PC1 and/or PC2 is shown in Additional file 1: Fig. S7.

### Associations between MISI and HIRI with postprandial plasma metabolites

We also examined associations of MISI and HIRI with postprandial plasma metabolite iAUCs. HIRI was associated with three of the 164 absolute postprandial metabolite responses, of which one in women only, and 17 of the 82 relative metabolite measures, of which 11 in women only (Additional file 1: Fig. S1). MISI was not significantly associated to any of the postprandial metabolite responses after adjustment for multiple testing.

As for the three absolute postprandial metabolite responses, HIRI was positively associated to postprandial TAG in S LDL (*p* = 0.043), XL HDL (*p* = 0.043), and S VLDL particles (*p* = 0.034), the latter in women only (Fig. 2). For the 17 relative metabolite responses, HIRI was positively associated to TAG% in S LDL, M LDL, and S HDL particles (all *p* = 0.043) (Fig. 3), and negatively associated to cholesterol and cholesteryl esters in IDL and fatty acid omega-6 fraction (all *p* = 0.043). In addition, in women only, HIRI was positively associated to postprandial MUFA%, TAG% in S and XS VLDL, and negatively to cholesterol % and free cholesterol % in S and XS VLDL, CE% in XS VLDL, and phospholipids % in S VLDL (Fig. 3).

## Discussion

We investigated fasting and postprandial plasma metabolite profiles in tissue-specific IR. To this end, we measured 164 plasma metabolites, including lipoproteins, apolipoproteins, cholesterol, TAG, ketone bodies, and amino acids, for four hours after a high-fat mixed meal in individuals with predominant muscle IR or liver IR. Compared to individuals with muscle IR, individuals with liver IR had greater postprandial increases in concentrations of very large and large VLDL particles and medium-sized LDL particles, and lower postprandial plasma concentrations of IDL particles, while fasting lipoprotein profiles did not differ between IR phenotypes. In addition, individuals with liver IR had greater postprandial increases in TAG in very large, large, and small VLDL particles, all LDL subclasses, and small HDL, compared to those with muscle IR. Furthermore, postprandial plasma SFA and MUFA were higher, and total FA consisted of a larger percentage of SFA, and a lower percentage of PUFA postprandially in liver compared to muscle IR.

Elevated postprandial total TAG concentrations in liver compared to muscle IR have been reported previously [6]. To our knowledge, this study is the first to examine circulating lipoprotein subclasses and their composition in response to a high-fat mixed meal in tissue-specific IR. Compared to muscle IR, liver IR was characterized by a larger postprandial increase in plasma particle concentrations of large and very large VLDL particles, which was paralleled by larger increases in the TAG and cholesterol content of these VLDL subclasses. This greater postprandial increase can result from higher VLDL production, reduced clearance, or both. Studies that measured VLDL kinetics using stable isotope tracers have previously shown that IR, as assessed by HOMA-IR, which mainly reflects hepatic IR, was associated with increased hepatic production of large, TAG-rich VLDL [27, 28]. Hence, the greater postprandial increase in large and very large VLDL particles that we observed in people with liver IR may be due to larger hepatic VLDL production. There may be several mechanistic explanations for the greater production of large and very large VLDL particles in liver IR. Firstly, in healthy, insulin-sensitive individuals, insulin can directly inhibit VLDL production, partly by promoting the hepatic degradation of ApoB [29–32]. Hence, in liver IR, impaired insulin-mediated suppression of VLDL assembly and secretion may contribute to elevated postprandial VLDL levels, while this suppression is likely better maintained in individuals with predominant muscle IR. Secondly, insulin can increase VLDL production by inducing de novo lipogenesis (DNL) via activation of sterol regulatory element binding protein-1c (SREBP-1c), thereby promoting lipid synthesis [3]. This hepatic insulin action appears to be (largely) preserved in liver IR [33]. Higher hepatic IR, as measured with the gold-standard hyperinsulinemic-euglycemic clamp method, has indeed been positively associated to the relative contribution of hepatic DNL to plasma TAG in TAG-rich lipoproteins, indicating elevated DNL [34]. In the present study, individuals with liver IR had greater insulin excursions in the first hour after the meal, which may have contributed to greater VLDL production by increased DNL.

In addition to elevated postprandial VLDL-TAG in liver IR, liver IR was also characterized by higher postprandial TAG content in smaller LDL and HDL particles. As this was not paralleled by larger increases in particle concentrations, these lipoprotein subclasses were likely enriched in TAG in liver IR. In line with this, HIRI was positively associated to the TAG fraction of total lipid content in the smaller LDL and HDL subclasses. A potential explanation might be enhanced transfer of TAG from TAG-rich large and very large VLDL particles to LDL and HDL particles by increased activity of the enzyme cholesteryl ester transfer protein (CETP). CETP facilitates the transfer of TAG from large VLDL to LDL and HDL in exchange for CE, resulting in TAG-enriched LDL and HDL [35]. CETP activity has been shown to be mainly determined by plasma TAG levels, rather than by IR [36, 37], which indicates that potentially increased CETP action in individuals with liver IR may be primarily attributable to the increased postprandial TAG in the circulation, and not to the hepatic IR itself.

Furthermore, liver IR was characterized by higher postprandial TAG fraction in IDL and smaller VLDL particles as compared to muscle IR. A higher TAG fraction in these particles, which are the VLDL remnants, may point towards reduced lipoprotein lipase (LPL)-mediated lipolysis of TAG in peripheral tissues in liver compared to muscle IR. IR has indeed been associated to lower LPL activity or expression in adipose tissue and skeletal muscle [38–40]. As far as we know, LPL activity has not been investigated in tissue-specific IR yet.

In liver compared to muscle IR, the postprandial plasma FA profile was characterized by greater total FA, SFA, and MUFA concentrations, a lower percentage of PUFA of total FA, and a higher percentage of SFA of total FA after mixed-meal ingestion. These results were independent of habitual dietary intake of fat, SFA, or LA, as assessed by FFQ. DNL produces mainly SFA, which can subsequently be desaturated to MUFA in the liver [41–43]. The higher early postprandial insulin response in individuals with liver IR may have promoted DNL as described above, thereby contributing to a greater postprandial increase in SFA. Recently, the proportion of SFA in VLDL has been reported to strongly correlate to the hepatic SFA fraction and, in turn, a higher hepatic SFA fraction to correlate to more severe hepatic IR [44]. Hence, higher hepatic SFA availability might also have contributed to the elevated postprandial plasma SFA in liver IR.

Using multivariate analysis of the postprandial metabolite curves, we additionally identified differences in postprandial metabolite curve shapes between the IR phenotypes for GlycA. GlycA is a relatively novel inflammatory biomarker that reflects the abundance of glycan groups of several acute-phase proteins [45]. Individuals with liver IR had larger postprandial GlycA increases than those with muscle IR. This difference may possibly be (partly) attributed to the observed greater postprandial TAG in liver as compared to muscle IR, since GlycA response to a mixed meal has recently been shown to strongly correlate to postprandial TAG peak [46].

Both liver and muscle IR have previously been associated with elevated fasting levels of the amino acids alanine, valine, and isoleucine, while liver IR, but not muscle IR has been additionally associated with higher circulating leucine and tyrosine, as well as lower circulating ketone bodies [5]. In the present study, we observed similar inverse associations between MISI and fasting plasma isoleucine and alanine, although these were no longer statistically significant after adjustment for multiple testing. Other associations could (also) not be replicated. An important explanation for these incongruencies may be differences in the study population: we selected individuals with predominant muscle or liver IR, thus excluding insulin-sensitive individuals and individuals with combined muscle and liver IR, resulting in a smaller range of MISI and HIRI. This may also explain why we found more metabolites to significantly differ between individuals with liver or muscle IR than in the associations with MISI and HIRI. In addition, the cohorts used by Vogelzangs et al. [5] were much larger (n = 634 and n = 540) than our study population.

It is well established that sex differences in lipid metabolism exist, which may contribute to differences in the aetiology of chronic cardiometabolic diseases between men and women [47]. Interestingly, we also observed sex differences, with a more pronounced link between liver IR and postprandial lipid profile in women compared to men. In line with this, women with liver IR had more VAT and lower whole-body insulin sensitivity compared to women with muscle IR, while these parameters did not differ between IR phenotypes in men. Although women generally have a more favourable plasma lipid profile than men, various studies indicate that in impaired metabolic health, i.e. obesity or T2DM, women have greater abnormalities in lipid and lipoprotein metabolism than men [48–53]. Similarly, hepatic IR has previously been associated with plasma lipid abnormalities in the fasting state—including elevated TAG, DAG, and BCAA—in women, and not in men [4, 5]. Lipid metabolism in women is also affected by sex hormones and menopausal state [47]. Most women in our study (84%) were postmenopausal, and the limited number of premenopausal women (n = 21) did not allow for performing stratified analyses. The observed sex differences in the relationship between tissue-specific IR and postprandial lipoprotein profile highlight the importance of taking sexual dimorphism into account and warrant further research to elucidate underlying mechanisms.

The lipoprotein profile we observed in liver compared to muscle IR—elevated postprandial TAG in larger VLDL, LDL, and smaller HDL particles—is common in IR and T2DM [30, 31, 54, 55]. Such a lipid profile is considered to be highly atherogenic and has consistently been associated with increased CVD risk [7–9, 56, 57]. Our findings show that this postprandial lipid profile is specifically related to liver IR, and less so to muscle IR, despite similar body fat percentage, liver fat, and whole-body insulin sensitivity, and lower systemic low-grade inflammation, as indicated by lower circulating CRP levels. Humans typically spend the majority of the day in the postprandial state due to frequent eating occasions. Even in healthy, insulin-sensitive individuals, plasma TAG levels progressively rise throughout the day upon repeated meal consumption, returning only to fasting levels during sleep [58]. Thus, individuals with liver IR may be at increased risk of developing cardiometabolic disease, compared to individuals with muscle IR.

Interestingly, the more unfavorable lipoprotein profile in liver compared to muscle IR was not observed in the fasting state. Differences between IR phenotypes only became apparent after challenging homeostasis with a high-fat mixed meal. In both individuals with liver and muscle IR, the majority had fasting TAG concentrations in the normal range (< 1.7 mmol/L): 72% in muscle IR and 64% in liver IR. In addition, a large majority was normoglycemic: 77% in muscle IR and 78% in liver IR. These findings thus indicate that liver IR, in particular, is accompanied by early perturbations in postprandial lipid metabolism that are not evident in the fasting state yet compared to muscle IR. Detection of metabolic perturbations at this early stage—before the onset of overt metabolic disease—provides an opportunity for timely prevention of progression to cardiometabolic disease by lifestyle interventions such as dietary modification, exercise, and weight loss.

A major strength of this study is the extensive metabolic profiling of tissue-specific IR in both the fasting and the postprandial state, thereby broadening and deepening the characterization of plasma lipid profiles in tissue-specific IR and providing more insights into the metabolic abnormalities that are related to muscle or liver IR. It is as of yet unknown whether dysregulated lipid metabolism is a cause or consequence of hepatic IR. Due to the cross-sectional design of this study, we cannot make any causal inferences about the nature of the observed relationship between tissue-specific IR and postprandial plasma lipid profiles. Another limitation of this study is the use of OGTT-derived measures for the assessment of tissue-specific IR. Postprandial glycemic and insulin responses are also affected by gastrointestinal factors such as gastric emptying and the incretin response [59]. Therefore, MISI and HIRI provide a less precise estimation of tissue-specific IR compared to the gold-standard two-step hyperinsulinemic-euglycemic clamp. Nevertheless, these indices have been validated against the gold-standard clamp [14] and we have previously shown that using these OGTT-derived measures, we could identify distinct metabolic phenotypes in various cohorts [4, 5, 60]. In addition, our study population consisted only of individuals with some degree of tissue-specific IR. Therefore, we cannot conclude anything on postprandial metabolic profiles in tissue-specific IR as compared to healthy, insulin-sensitive controls. Finally, we sampled blood until four hours after consumption of the meal, at which many plasma lipids are at their peak. Future studies would benefit from longer sampling times until 6–8 h post-meal to allow for examination of the (rate of) return to fasting levels or the effects of a second meal.

## Conclusion

In conclusion, individuals with liver IR or muscle IR have distinct postprandial plasma metabolite responses after a mixed meal, despite similar fasting metabolite profiles, body fat percentage, and whole-body insulin sensitivity. Liver IR was characterized by greater postprandial increases in larger VLDL particles, as well as in TAG in the larger VLDL and the smaller LDL and HDL subclasses, compared to muscle IR, which points towards more impaired hepatic lipid metabolism in liver compared to muscle IR. Therefore, improving postprandial lipid metabolism with lifestyle modifications to prevent the development of cardiometabolic disease may be particularly important for individuals with predominant liver IR.

### Supplementary Information

**Additional file 1: **Additional information on methods of RM-ASCA + analysis. **Figure S1.** Flow diagram of the number of fasting and postprandial metabolites that were significantly different between individuals with liver or muscle IR. **Figure S2.** Fasting plasma fatty acids, fatty acid ratios, (branched-chain) amino acids, and ketone bodies in liver versus muscle IR. **Figure S3.** Postprandial (iAUC) lipoprotein particle concentrations, cholesterol, and cholesteryl esters in liver IR versus muscle IR. **Figure S4.** Responses of plasma lipoprotein particles, lipoprotein triglycerides, and lipoprotein lipid composition to a high-fat mixed meal in muscle and liver IR. **Figure S5.** Responses of plasma fatty acids and fatty acid fractions to a high-fat mixed meal in muscle and liver IR. **Figure S6.** Selection of plasma metabolite responses to a high-fat mixed meal that were identified to have differential temporal patterns between liver and muscle IR in RM-ASCA + analysis. **Figure S7.** Score trajectory and loading plots of the difference in postprandial metabolite responses in liver IR compared to muscle IR using RM-ASCA + analysis.

**Additional file 2: Table S1.** Demographic, clinical and metabolic characteristics of the study population according to IR phenotype and sex. **Table S2.** Fasting plasma metabolites in muscle (muscle IR) and liver insulin resistance (liver IR). **Table S3.** Postprandial plasma metabolites (iAUC) in muscle (muscle IR) and liver insulin resistance (liver IR). **Table S4.** Postprandial plasma metabolite responses in muscle (muscle IR) and liver insulin resistance (liver IR).

## Data Availability

Data are available from the authors upon request.

## Abbreviations

BCAA: Branched-chain amino acids
CETP: Cholesteryl ester transfer protein
CRP: C-reactive protein
CVD: Cardiovascular disease
DAG: Diacylglycerol
DHD15-index: Dutch Healthy Diet index 2015
DNL: De novo Lipogenesis
DXA: Dual-energy X-ray absorptiometry
ELISA: Enzyme-linked immunoassay
FDR: False discovery rate
FFQ: Food frequency questionnaire
HDL: High-density lipoprotein
HIRI: Hepatic insulin resistance index
HOMA- β: Homeostasis model assessment of β-cell function
HOMA-IR: Homeostasis model assessment of insulin resistance
iAUC: Incremental area under the curve
IDL: Intermediate-density lipoprotein
IFG: Impaired fasting glucose
IGT: Impaired glucose tolerance
IR: Insulin resistance
LA: Linoleic acid
LDL: Low-density lipoprotein
LPL: Lipoprotein lipase
MISI: Muscle insulin sensitivity index
MRI: Magnetic resonance imaging
MUFA: Monounsaturated fatty acids
NEFA: Non-esterified fatty acids
NGT: Normal glucose tolerance
NMR: Nuclear magnetic resonance
OGTT: Oral glucose tolerance test
PUFA: Polyunsaturated fatty acids
SFA: Saturated fatty acids
SREBP-1c: Sterol regulatory element binding protein-1c
T2DM: Type 2 diabetes
TAG: Triacylglycerol
VAT: Visceral adipose tissue
VLDL: Very-low-density lipoprotein
