# Supplementary material for: Hepatic insulin resistance and muscle insulin resistance are characterized by distinct postprandial plasma metabolite profiles: a cross-sectional study
Source: Cardiovasc Diabetol. 2024 Mar 16;23:97. doi: 10.1186/s12933-024-02188-0 (PMC10944619; doi:10.1186/s12933-024-02188-0)
Supplement: Supplementary file 1 — Additional file 1: Additional information on methods of RM-ASCA + analysis. Figure S1. Flow diagram of the number of fasting and postprandial metabolites that were significantly different between individuals with liver or muscle IR. Figure S2. Fasting plasma fatty acids, fatty acid ratios, (branched-chain) amino acids, and ketone bodies in liver versus muscle IR. Figure S3. Postprandial (iAUC) lipoprotein particle concentrations, cholesterol, and cholesteryl esters in liver IR versus muscle IR. Figure S4. Responses of plasma lipoprotein particles, lipoprotein triglycerides, and lipoprotein lipid composition to a high-fat mixed meal in muscle and liver IR. Figure S5. Responses of plasma fatty acids and fatty acid fractions to a high-fat mixed meal in muscle and liver IR. Figure S6. Selection of plasma metabolite responses to a high-fat mixed meal that were identified to have differential temporal patterns between liver and muscle IR in RM-ASCA + analysis. Figure S7. Score trajectory and loading plots of the difference in postprandial metabolite responses in liver IR compared to muscle IR using RM-ASCA + analysis. [file 12933_2024_2188_MOESM1_ESM.pdf]

Supplementary information on methods of RM-ASCA+ analysis

We performed multivariate analysis to compare the shapes of the metabolite responses between IR phenotypes via RM-ASCA+[1]. This approach allows the quantification of multivariate outcomes in studies with repeated measurements while accounting for proper within-subject dependency structures, such as temporal dependence and interrelatedness of metabolites. The metabolites concentration/ratio y at timepoint t in subject i with phenotype h were modelled according to the linear mixed model:

$$y_{\ iht} = (\beta_0 + \gamma_i) + \beta \quad {}_x + \beta \qquad + \beta \qquad + \beta \qquad + \beta \qquad +$$

$$+\beta_1 x_1(t) g_h + \beta_2 x_2(t) g_h + \epsilon_{iht}$$

where β are the fixed effects coefficients for intercept, sex, BMI, WHR, center, age, and time-phenotype interaction (β_1, β_2) with corresponding basis x_{1-2} of a natural cubic spline with two degrees of freedom[2]. In order to adjust for fasting metabolite concentrations, the main effect for phenotype is not included in the models. γ are random intercepts, ϵ is the residual term, and g the reference coded indicator variable for phenotype h, with muscle IR as reference. We assume, that $\gamma_1 \dots \gamma_I$ and $\boldsymbol{\epsilon}_1 \dots \boldsymbol{\epsilon}_I$ are independent, $\gamma_{i0} \sim \mathcal{N}(0, \sigma_\gamma^2)$, and $\epsilon_{iht} \sim \mathcal{N}(0, \sigma_\epsilon^2)$. The univariate metabolite model residuals were checked for heteroskedasticity and trend in the residuals. Four metabolites were log-transformed prior to analysis to ensure homoskedasticity of the residuals.

In RM-ASCA+, the metabolite responses y_{iht}, the corresponding coefficients β, and random variables γ were then combined into multivariate effect matrices and analysed as described in detail elsewhere[1]. Here, the effect matrix of interest is composed of the multivariate time-phenotype interaction terms $\sum_{k=1}^{2} \beta_k x_k(t) g_h$ related to the effect of phenotype. In other words, we analysed the group (i.e. phenotype) difference in within-group change in metabolite concentration from fasting (i.e. the meal effect), expressed as deviations from the reference group (muscle IR phenotype).

Model validation was carried out via nonparametric bootstrapping to construct 95% confidence intervals for the score and loadings associated with the effect matrix for time-phenotype interaction. The procedure is described in detail elsewhere[3].

1. Erdős B, Westerhuis JA, Adriaens ME, O'Donovan SD, Xie R, Singh-Povel CM, et al. Analysis of high-dimensional metabolomics data with complex temporal dynamics using RM-ASCA+. PLOS Computational Biology. 2023;19(6):e1011221.
2. Hastie TJ. Statistical Models in S, Chapter 7: Generalized additive models. 1st ed. ed: Routledge; 1992.
3. Madssen TS, Giskeødegård GF, Smilde AK, Westerhuis JA. Repeated measures ASCA+ for analysis of longitudinal intervention studies with multivariate outcome data. PLOS Computational Biology. 2021;17(11):e1009585.

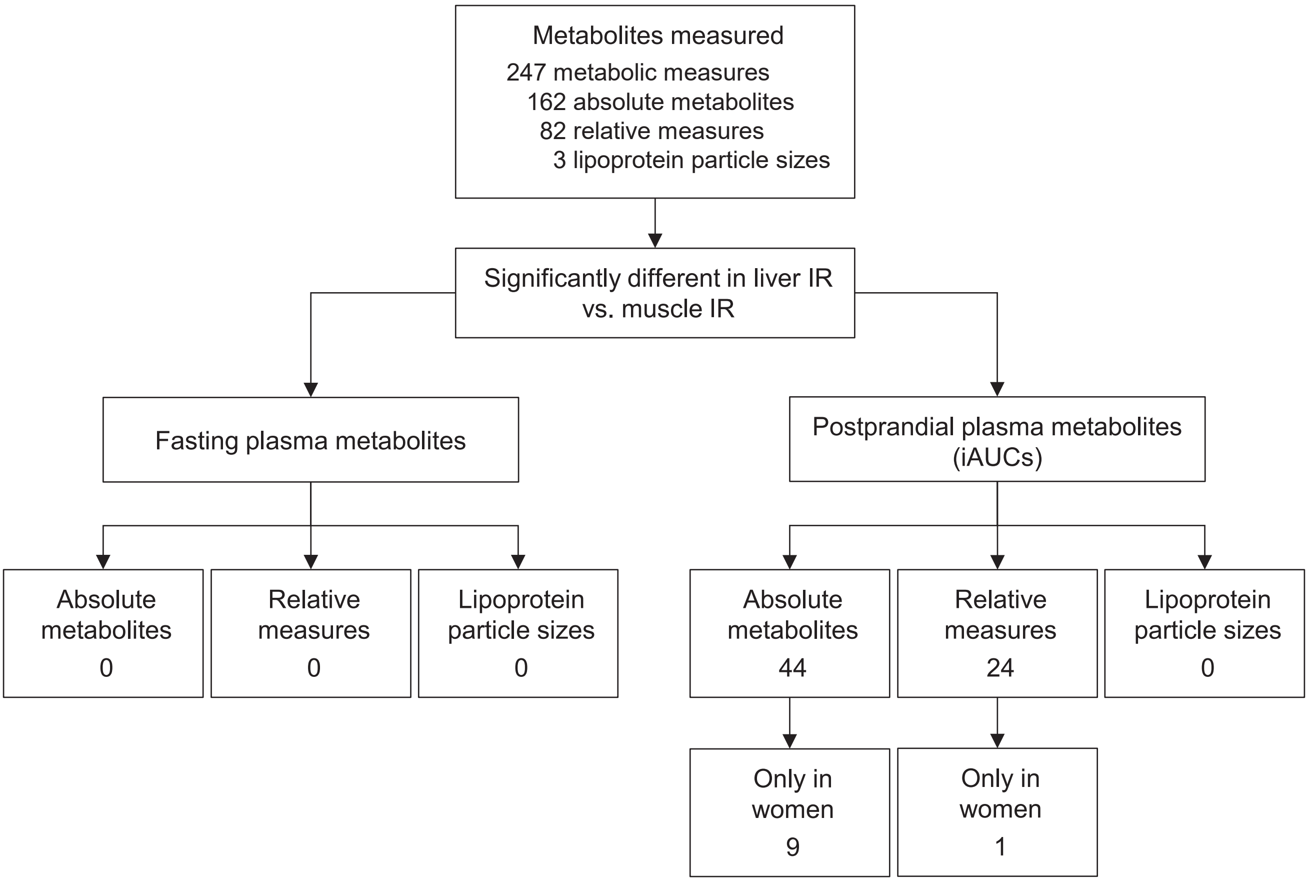

Figure S1. Flow diagram of the number of fasting and postprandial metabolites that were significantly different between individuals with muscle or liver IR. Differences between liver IR and muscle IR were tested using ANCOVA with adjustment for age, sex, centre, BMI, and waist-to-hip ratio. P-values were adjusted for multiple testing using the Benjamini-Hochberg method.

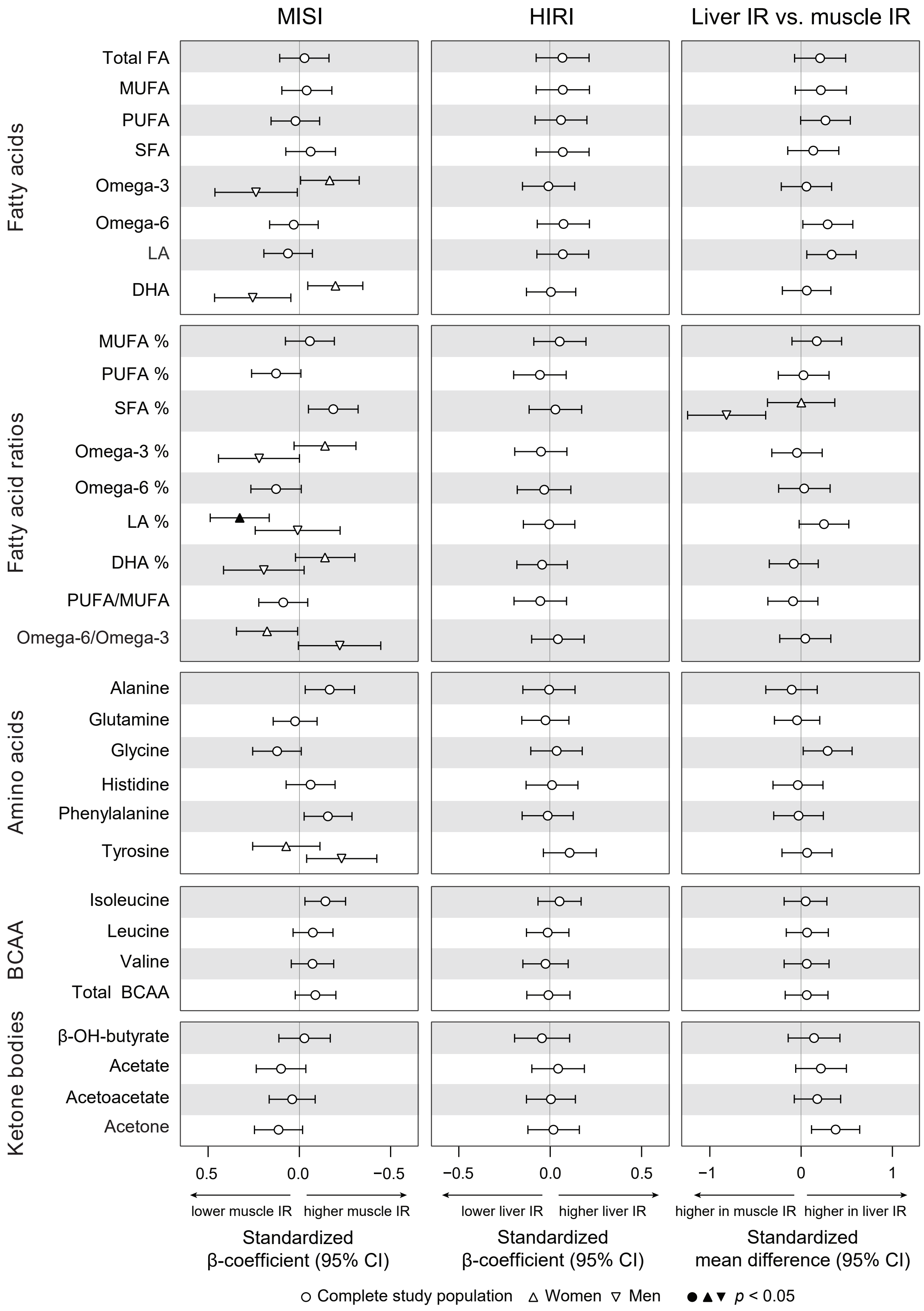

Figure S2. Fasting plasma fatty acids, fatty acid ratios, (branched-chain) amino acids, and ketone bodies in muscle and liver IR. Left: associations of MISI with fasting plasma metabolites. Middle: associations of HIRI with fasting plasma metabolites. Right: fasting plasma metabolite in muscle compared to liver IR. Associations between MISI/HIRI and plasma metabolites were tested using linear regression analyses with adjustment for age, sex, center, BMI, waist-to-hip ratio and HIRI/MISI. Differences between muscle and liver IR were tested using ANCOVA with adjustment for age, sex, center, BMI, and waist-to-hip ratio. *P*-values were adjusted for multiple testing using the Benjamini-Hochberg method.

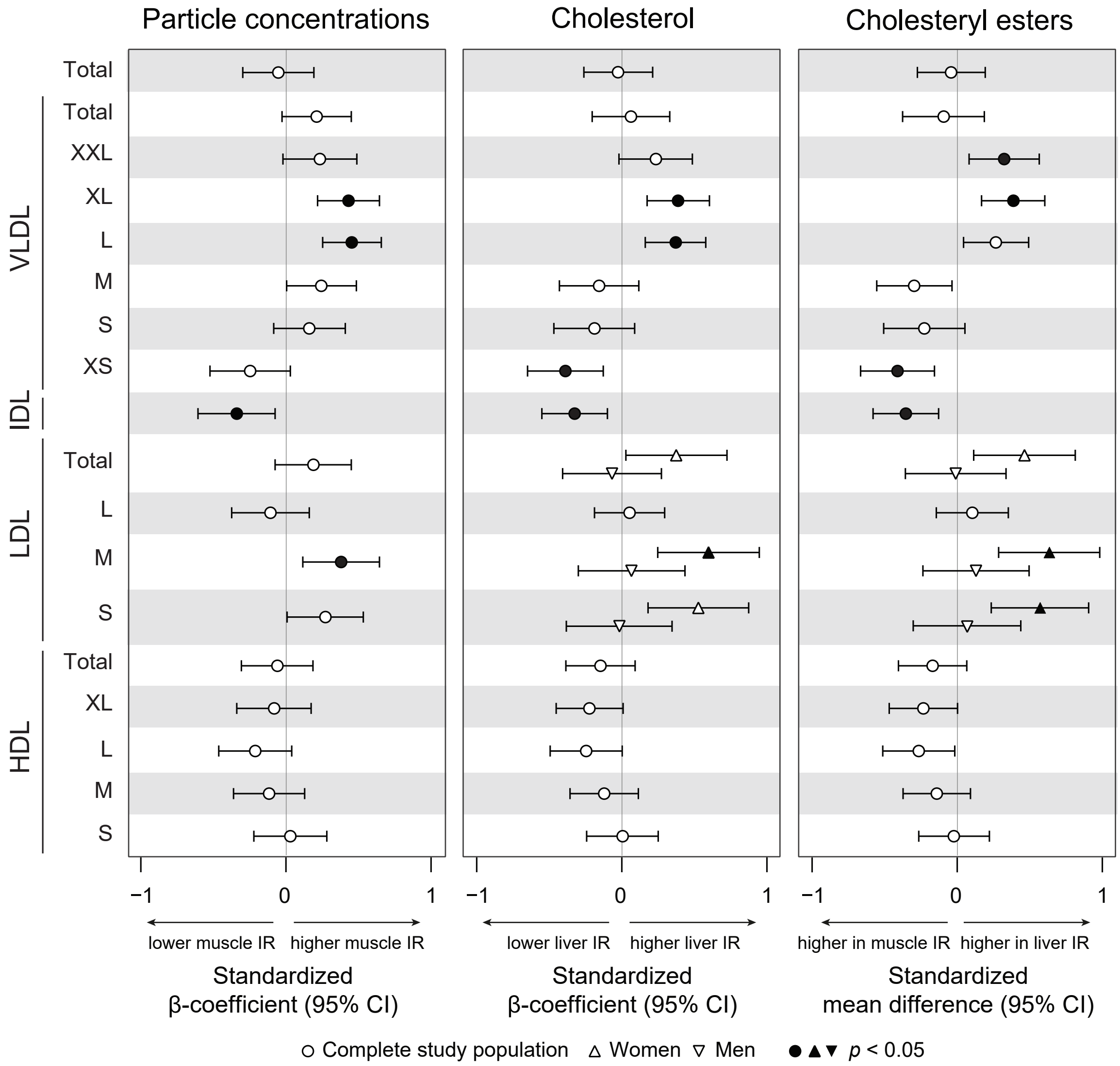

Figure S3. Postprandial (iAUC) lipoprotein particle concentrations, cholesterol, and cholesteryl esters in muscle and liver IR. Differences between muscle and liver IR were tested using ANCOVA with adjustment for age, sex, center, BMI, and waist-to-hip ratio. *P*-values were adjusted for multiple testing using the Benjamini-Hochberg method.

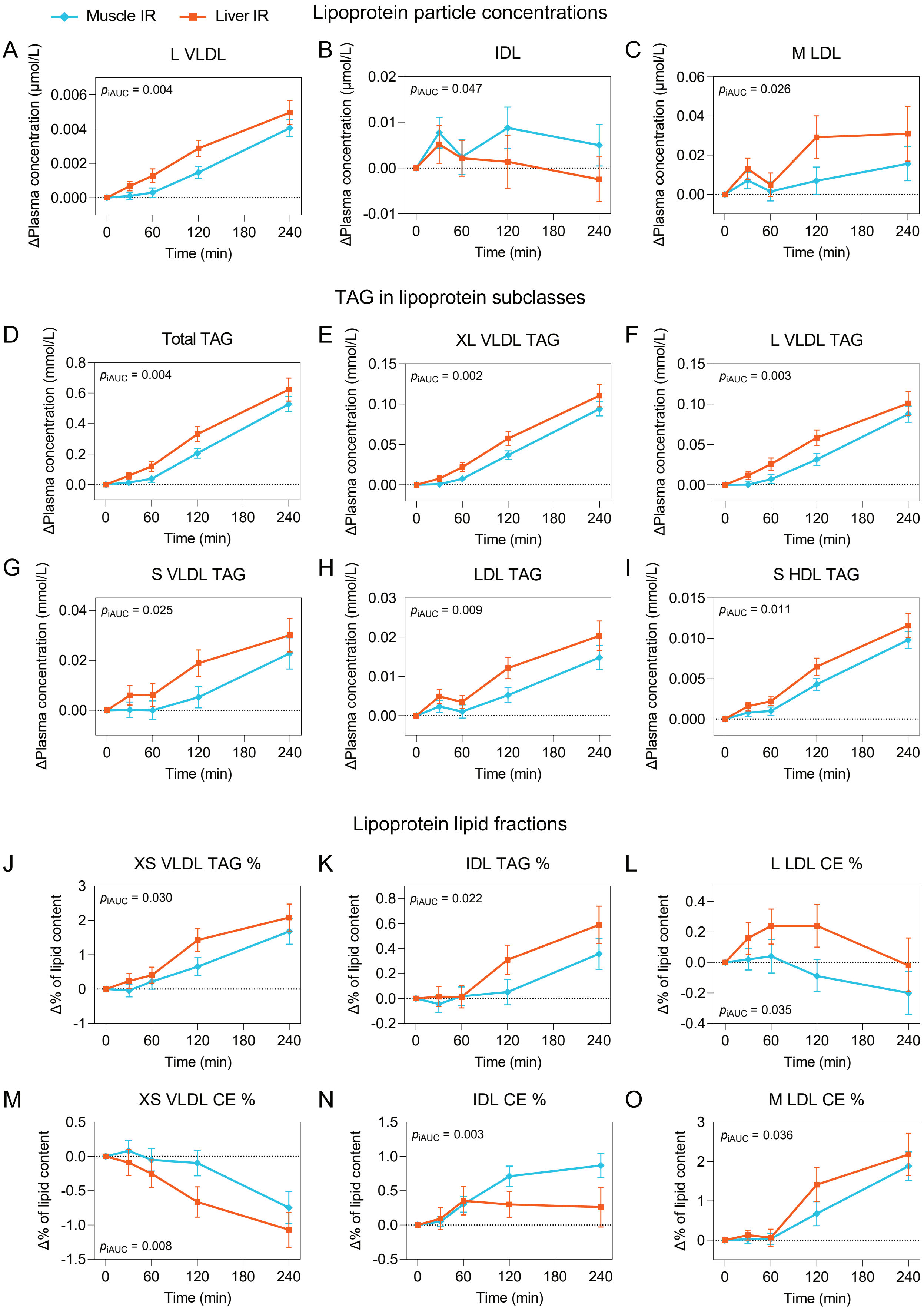

Figure S4. Responses of plasma lipoprotein particles, lipoprotein triglycerides, and lipoprotein lipid composition to a high-fat mixed meal in muscle and liver IR. Responses were defined as change from fasting (value at postprandial timepoint – fasting value) and data are shown as means with 95% confidence intervals. Differences in incremental area under the curves (iAUCs) between liver IR and muscle IR were tested using ANCOVA with adjustment for age, sex, center, BMI, and waist-to-hip ratio. *P*-values were adjusted for multiple testing using the Benjamini-Hochberg method.

Fatty acids and fatty acid fractions

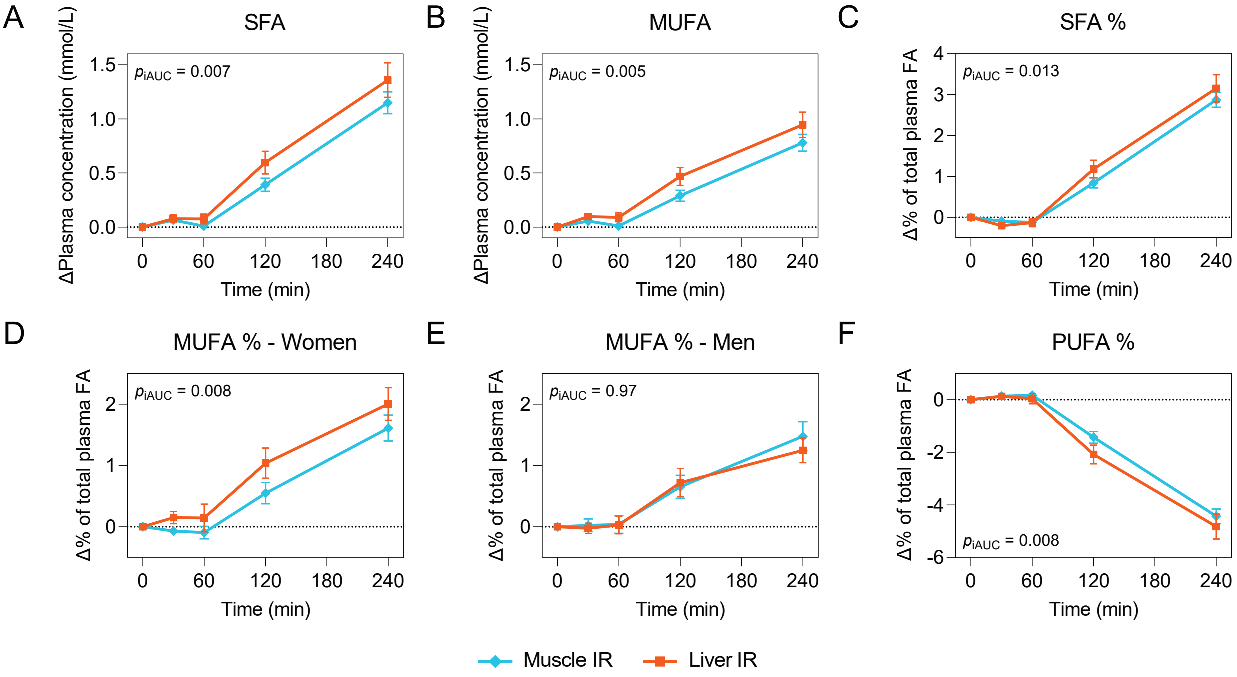

Figure S5. Responses of plasma fatty acids and fatty acid fractions to a high-fat mixed meal in muscle and liver IR. Responses were defined as change from fasting (value at postprandial timepoint – fasting value) and data are shown as means with 95% confidence intervals. Differences in incremental area under the curves (iAUCs) between liver IR and muscle IR were tested using ANCOVA with adjustment for age, sex, center, BMI, and waist-to-hip ratio. *P*-values were adjusted for multiple testing using the Benjamini-Hochberg method.

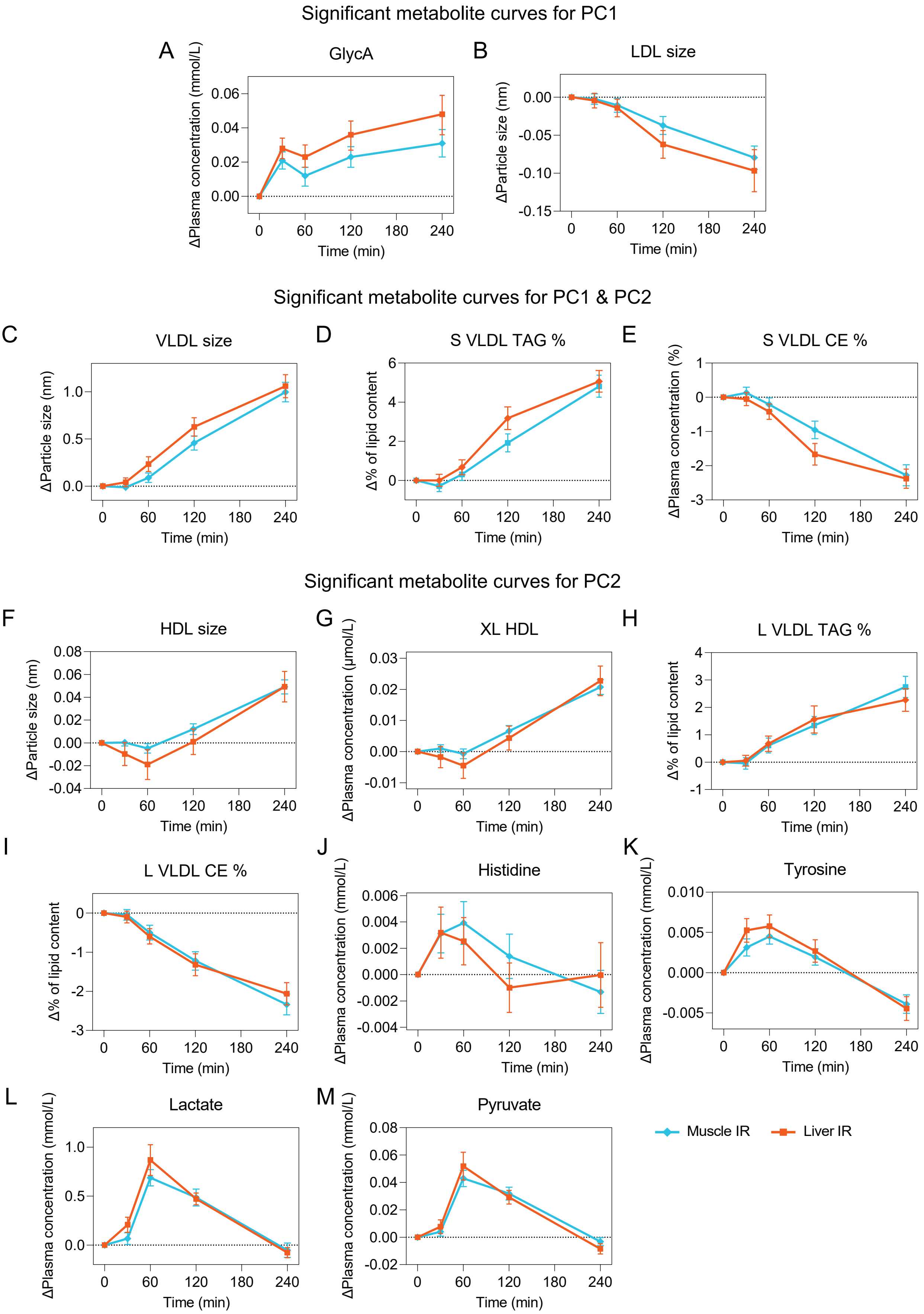

Figure S6. Selection of plasma metabolite responses to a high-fat mixed meal that were identified to have differential temporal patterns between liver and muscle IR in RM-ASCA+ analysis. Responses were defined as change from fasting (value at postprandial timepoint – fasting value) and data are shown as means with 95% confidence intervals.

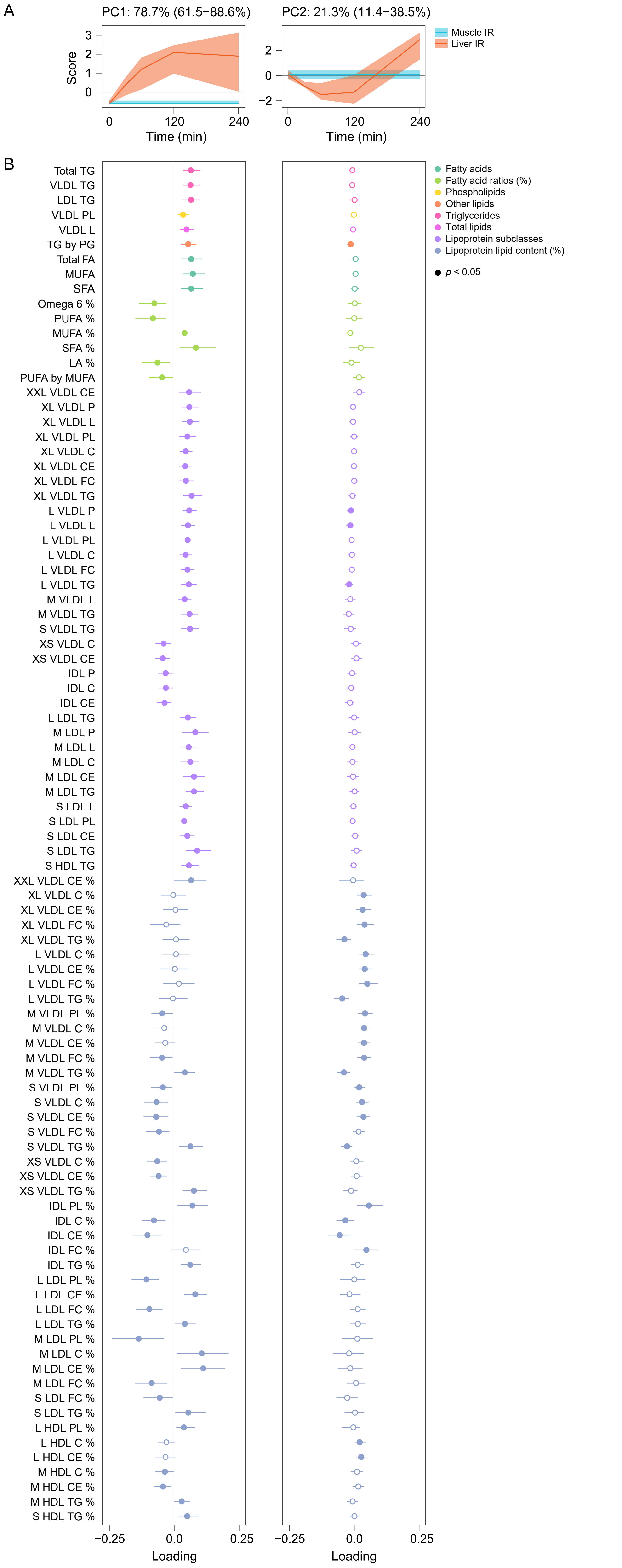

Figure S7. Score trajectory (A) and loading (B) plots of the difference in postprandial metabolite responses in liver IR compared to muscle IR using RM-ASCA+ analysis. (A) Scores containing the predominant patterns over time for PC1 and PC2. Bootstrapped 95% confidence intervals are shown as shaded area for the scores. (B) Loading plots of metabolites with a significant loading for PC1 and/or PC2, excluding those shown in Fig. 5. The loadings show the association of the scores with the metabolite responses. A positive loading indicates a positive association between the metabolite response and the score (pattern) as shown in this figure, while a negative loading indicates a negative association between the metabolite response and the score (pattern) as shown in this figure, i.e. the inverse of the depicted pattern. Bootstrapped 95% confidence intervals are shown as error bars for the loadings. Filled dots indicate $p < 0.05$.
